# Strategies to prevent central line-associated bloodstream infections in acute-care hospitals: 2022 Update

**DOI:** 10.1017/ice.2022.87

**Published:** 2022-05

**Authors:** Niccolò Buetti, Jonas Marschall, Marci Drees, Mohamad G. Fakih, Lynn Hadaway, Lisa L. Maragakis, Elizabeth Monsees, Shannon Novosad, Naomi P. O’Grady, Mark E. Rupp, Joshua Wolf, Deborah Yokoe, Leonard A. Mermel

**Affiliations:** 1 Infection Control Programme, University of Geneva Hospitals and Faculty of Medicine, Geneva, Switzerland; 2 University of Paris, Paris, France; 3 Department of Infectious Diseases, Bern University Hospital and University of Bern, Bern, Switzerland; 4 Division of Infectious Diseases, Department of Medicine, Washington University School of Medicine, St. Louis, Missouri, United States; 5 ChristianaCare, Wilmington, Delaware, United States; 6 Sidney Kimmel Medical College at Thomas Jefferson University, Philadelphia, Pennsylvania, United States; 7 Ascension Healthcare and Wayne State University School of Medicine, Detroit, Michigan, United States; 8 Lynn Hadaway Associates, Milner, Georgia, United States; 9 Johns Hopkins University School of Medicine, Baltimore, Maryland, United States; 10 Children’s Mercy Hospital, Kansas City, Missouri, United States; 11 University of Missouri–Kansas City School of Medicine, Kansas City, Missouri, United States,; 12 Division of Healthcare Quality Promotion, Centers for Disease Control and Prevention, Atlanta, Georgia, United States; 13 National Institutes of Health, Bethesda, Maryland, United States; 14 University of Nebraska Medical Center, Omaha, Nebraska, United States; 15 Department of Infectious Diseases, St. Jude Children’s Research Hospital, Memphis, Tennessee, United States; 16 Department of Pediatrics, University of Tennessee Health Science Center, Memphis, Tennessee, United States; 17 University of California–San Francisco, San Francisco, California, United States; 18 Warren Alpert Medical School of Brown University, Providence, Rhode Island, United States; 19 Rhode Island Hospital, Providence, Rhode Island, United States

## Purpose

Previously published guidelines provide comprehensive recommendations for detecting and preventing healthcare-associated infections (HAIs). The intent of this document is to highlight practical recommendations in a concise format designed to assist acute-care hospitals in implementing and prioritizing their central line-associated bloodstream infection (CLABSI) prevention efforts. This document updates the *Strategies to Prevent Central Line-Associated Bloodstream Infections in Acute-Care Hospitals* published in 2014.^1^ This expert guidance document is sponsored by the Society for Healthcare Epidemiology of America (SHEA). It is the product of a collaborative effort led by SHEA, the Infectious Diseases Society of America (IDSA), the Association for Professionals in Infection Control and Epidemiology (APIC), the American Hospital Association (AHA), and The Joint Commission, with major contributions from representatives of a number of organizations and societies with content expertise.

## Summary of major changes

This section lists major changes from the *Strategies to Prevent Central Line-Associated Bloodstream Infections in Acute-Care Hospitals: 2014 Update*,^1^ including recommendations that have been added, removed, or altered. Recommendations are categorized as essential practices that should be adopted by all acute-care hospitals (in 2014 these were “basic practices,” renamed to highlight their importance as foundational for hospitals’ HAI prevention programs) or additional approaches that can be considered for use in locations and/or populations within hospitals when CLABSIs are not controlled after implementation of essential practices (in 2014 these were “special approaches”). See Table 1 for a complete summary of the recommendations contained in this document.

**Table 1.** Summary of Recommendations to Prevent CLABSI

Essential Practices
*Before insertion* 1. Provide easy access to an evidence-based list of indications for CVC use to minimize unnecessary CVC placement (Quality of Evidence: LOW) 2. Require education and competency assessment of HCP involved in insertion, care, and maintenance of CVCs about CLABSI prevention (Quality of Evidence: MODERATE)^74–78^ 3. Bathe ICU patients aged >2 months with a chlorhexidine preparation on a daily basis (Quality of Evidence: HIGH)^86–90^ *At insertion* 1. In ICU and non-ICU settings, a facility should have a process in place, such as a checklist, to ensure adherence to infection prevention practices at the time of CVC insertion (Quality of Evidence: MODERATE)^101^ 2. Perform hand hygiene prior to catheter insertion or manipulation (Quality of Evidence: MODERATE)^102–107^ 3. The subclavian site is preferred to reduce infectious complications when the catheter is placed in the ICU setting (Quality of Evidence: HIGH)^33,37,108–110^ 4. Use an all-inclusive catheter cart or kit (Quality of Evidence: MODERATE)^118^ 5. Use ultrasound guidance for catheter insertion (Quality of Evidence: HIGH)^119,120^ 6. Use maximum sterile barrier precautions during CVC insertion (Quality of Evidence: MODERATE)^123–128^ 7. Use an alcoholic chlorhexidine antiseptic for skin preparation (Quality of Evidence: HIGH)^42,129–134^ *After insertion* 1. Ensure appropriate nurse-to-patient ratio and limit use of float nurses in ICUs (Quality of Evidence: HIGH)^34,35^ 2. Use chlorhexidine-containing dressings for CVCs in patients over 2 months of age (Quality of Evidence: HIGH)^45,135–142^ 3. For non-tunneled CVCs in adults and children, change transparent dressings and perform site care with a chlorhexidine-based antiseptic at least every 7 days or immediately if the dressing is soiled, loose, or damp. Change gauze dressings every 2 days or earlier if the dressing is soiled, loose, or damp (Quality of Evidence: MODERATE)^145–148^ 4. Disinfect catheter hubs, needleless connectors, and injection ports before accessing the catheter (Quality of Evidence: MODERATE)^150–154^ 5. Remove nonessential catheters (Quality of Evidence: MODERATE) 6. Routine replacement of administration sets not used for blood, blood products, or lipid formulations can be performed at intervals up to 7 days (Quality of Evidence: HIGH)^164^ 7. Perform surveillance for CLABSI in ICU and non-ICU settings (Quality of Evidence: HIGH)^13,165,166^
**Additional Approaches**
1. Use antiseptic- or antimicrobial-impregnated CVCs (Quality of Evidence: HIGH in adult patients^38,39,169–171^ and Quality of Evidence: MODERATE in pediatric patients)^172,173^ 2. Use antimicrobial lock therapy for long-term CVCs (Quality of Evidence: HIGH)^177–184^ 3. Use recombinant tissue plasminogen activating factor (rt-PA) once weekly after hemodialysis in patients undergoing hemodialysis through a CVC (Quality of Evidence: HIGH)^192^ 4. Utilize infusion or vascular access teams for reducing CLABSI rates (Quality of Evidence: LOW)^193,194^ 5. Use antimicrobial ointments for hemodialysis catheter insertion sites (Quality of Evidence: HIGH)^197–201^ 6. Use an antiseptic-containing hub/connector cap/port protector to cover connectors (Quality of Evidence: MODERATE)^202–208^
**Approaches that Should Not Be Considered a Routine Part of CLABSI Prevention**
1. Do not use antimicrobial prophylaxis for short-term or tunneled catheter insertion or while catheters are *in situ* (Quality of Evidence: HIGH)^209–213^ 2. Do not routinely replace CVCs or arterial catheters (Quality of Evidence: HIGH)^214^
**Unresolved Issues**
1. Routine use of needleless connectors as a CLABSI prevention strategy before an assessment of risks, benefits, and education regarding proper use^215–219^ 2. Surveillance of other types of catheters (eg, peripheral arterial or peripheral venous catheters)^11,21,22^ 3. Standard, nonantimicrobial transparent dressings and CLABSI risk. 4. The impact of using chlorhexidine-based products on bacterial resistance to chlorhexidine 5. Sutureless securement 6. Impact of silver zeolite-impregnated umbilical catheters in preterm infants (applicable in countries where it is approved for use in children)^227^ 7. Necessity of mechanical disinfection of a catheter hub, needleless connector, and injection port before accessing the catheter when antiseptic-containing caps are being used

Note. CLABSI, central line-associated bloodstream infection; CVC, central venous catheter; HCP, healthcare personnel; ICU, intensive care unit.

### Essential practices

- The subclavian vein is considered the preferable site for central venous catheter (CVC) insertion in the intensive care setting to reduce infectious complications. Previously, the primary recommendation was to avoid the femoral vein for access. Although this remains valid, it has been replaced by a positively formulated recommendation regarding the subclavian site.
- The recommendation to use ultrasound guidance for catheter insertion is backed by better evidence than was available previously; however, the procedure itself may jeopardize the strict observation of sterile technique.
- The use of chlorhexidine-containing dressings is now considered an “essential practice”; in the past, it was listed under special approaches that should only be employed if CLABSI rates remain high despite the implementation of basic practices.
- Routine replacement of administration sets not used for blood, blood products, or lipid formulations can be performed at intervals of up to 7 days. Previously, this interval was no longer than 4 days.

### Additional approaches

- Antimicrobial ointment for the catheter site, which is geared toward the population of hemodialysis patients, has been moved to “additional practices” given the focus on a specific population.
- Despite currently being supported by high-level evidence, antiseptic-containing caps remain an “additional practice” because they are not considered superior to the manual disinfection, an essential practice.
- The importance of infusion teams has been highlighted by listing it under “additional practices” (previously considered unresolved).
- Sutureless securement of catheters was not discussed in the previous version of this section.

## Intended use

This document was developed following the process outlined in the *Handbook for SHEA-Sponsored Guidelines and Expert Guidance Documents*.^2^ No guideline or expert guidance document can anticipate all clinical situations, and this document is not meant to be a substitute for individual clinical judgment by qualified professionals.

This document is based on a synthesis of evidence, theoretical rationale, current practices, practical considerations, writing-group consensus, and consideration of potential harm, where applicable. A summary list of recommendations is provided along with their relevant rationales (see Table 1).

## Methods

SHEA recruited 3 subject-matter experts in the prevention of CLABSI to lead the panel of members representing the Compendium partnering organizations: SHEA, the Infectious Diseases Society of America (IDSA), the Association for Professionals in Infection Control and Epidemiology (APIC), the American Hospital Association (AHA), and The Joint Commission, as well as representation by the Centers for Disease Control and Prevention (CDC).

SHEA utilized a consultant medical librarian, who worked with each panel to develop a comprehensive search strategy for PubMed and Embase (January 2012–July 2019; updated to August 2021). Articles’ abstracts were reviewed by panel members in a double-blind fashion using the abstract management software, Covidence (Melbourne, Australia), and subsequently reviewed as full text. The Compendium Lead Authors group voted to update the literature findings, and the librarian reran the search to update it to August 2021. Panel members reviewed the abstracts of these articles via Covidence and incorporated relevant references.

Recommendations resulting from this literature review process were classified based on the quality of evidence and the balance between desirable and potential for undesirable effects of various interventions (see Table 2). Panel members met via video conference to discuss literature findings; recommendations; quality of evidence for these recommendations; and classification as essential practices, additional approaches, or unresolved issues. Panel members reviewed and approved the document and its recommendations.

**Table 2.** Quality of Evidence^a^

Category	Definition
HIGH	Highly confident that the true effect lies close to that of the estimated size and direction of the effect. Evidence is rated as high quality when there are a wide range of studies with no major limitations, there is little variation between studies, and the summary estimate has a narrow confidence interval.
MODERATE	The true effect is likely to be close to the estimated size and direction of the effect, but there is a possibility that it is substantially different. Evidence is rated as moderate quality when there are only a few studies and some have limitations but not major flaws, there is some variation between studies, and/or the confidence interval of the summary estimate is wide.
LOW	The true effect may be substantially different from the estimated size and direction of the effect. Evidence is rated as low quality when supporting studies have major flaws, there is important variation between studies, the confidence interval of the summary estimate is very wide, and/or there are no rigorous studies.

a Based on the CDC Healthcare Infection Control Practices Advisory Committee (HICPAC) “Update to the Centers for Disease Control and Prevention and the Healthcare Infection Control Practices Advisory Committee Recommendations Categorization Scheme for Infection Control and Prevention Guideline Recommendations” (October 2019), the Grades of Recommendation, Assessment, Development, and Evaluation (GRADE),^265^ and the Canadian Task Force on Preventive Health Care.^266^

The Compendium Expert Panel, made up of members with broad healthcare epidemiology and infection prevention expertise, reviewed the draft manuscript after consensus had been reached by writing panel members.

Following review and approval by the Expert Panel, the 5 partnering organizations, stakeholder organizations, and the CDC reviewed the document. Prior to dissemination, the guidance document was reviewed and approved by the SHEA Guidelines Committee, the IDSA Standards and Practice Guidelines Committee, and the Boards of SHEA, IDSA, APIC, AHA, and The Joint Commission.

All panel members complied with SHEA and IDSA policies on conflict-of-interest disclosure.

## Section 1: Rationale and statements of concern

### Burden of outcomes associated with hospital-acquired CLABSI

1. Increased length of hospital stay^3–6^
2. Increased cost. The adjusted variable costs for patients with CLABSI were $32,000 (2010 US dollars) higher on average than for patients without CLABSI^7^
3. Increased morbidity and mortality^8^

### Risk factors for CLABSI

1. Patients at risk for CLABSI in acute-care facilities are those with a CVC in place:
  - Intensive care unit (ICU) population: The risk of CLABSI in ICU patients is high. Reasons for this include the frequent insertion of multiple catheters^9,10^; the use of specific types of catheters that are almost exclusively inserted in ICU patients and associated with substantial risk (eg, pulmonary artery catheters with catheter introducers); and the fact that catheters are frequently placed in emergency circumstances, repeatedly accessed each day, and often needed for extended periods.^11,12^
  - Non-ICU population: Although the primary focus of attention over the last 20 years has been the ICU setting, most CLABSIs occur in hospital units outside the ICU or in outpatients.^13–17^
2. Infection prevention and control efforts should include other vulnerable populations such as patients receiving hemodialysis through catheters,^18^ intraoperative patients,^19^ and oncology patients.^20^
3. In addition to CVCs, short-term peripheral catheters,^21^ peripherally inserted central venous catheters (PICCs), midline catheters, and peripheral arterial catheters also carry a risk of infection.^22^
4. Independent risk factors for CLABSI (in at least 2 published studies)^23–45^
  - Prolonged hospitalization before catheterization
  - Prolonged duration of catheterization
  - Heavy microbial colonization at insertion site
  - Heavy microbial colonization of the catheter hub
  - Multilumen catheters
  - Concurrent catheters
  - Neutropenia
  - Body mass index (BMI) >40
  - Prematurity (ie, early gestational age)
  - Reduced nurse-to-patient ratio in the ICU
  - Parenteral nutrition
  - Substandard catheter care (eg, excessive manipulation of the catheter)
  - Transfusion of blood products (in children)

## Section 2: Background on detection of CLABSI

### Surveillance methods and definitions for CLABSI

1. Use consistent surveillance methods and definitions to allow comparison to benchmark data.
2. Refer to the *National Healthcare Safety Network* (*NHSN) Patient Safety Component Manual* for information on the appropriate surveillance methodology, including information about blood specimen collection and surveillance definitions of CLABSIs. The relevant chapter of the manual is “Chapter 4: Bloodstream Infection Event (Central Line-Associated Bloodstream Infection and Non-Central Line-Associated Bloodstream Infection).”^46^
  - Recent data suggest that interrater reliability using NHSN definitions is lower than expected.^47–50^ This may also affect the reliability of public reporting.
  - The NHSN surveillance definition for CLABSI is different than the clinical definition for catheter-related bloodstream infection (CRBSI). The latter is subject to various factors (eg, laboratory capabilities, catheter removal, and submitting the catheter tip for culture).^51^ The evidence presented here includes studies that used either CLABSI or CRBSI as an outcome measure and the lesser accuracy of CLABSI may impact the validity of the evidence.

## Section 3: Background on prevention of CLABSI

### Summary of existing guidelines and recommendations

1. Several governmental, public health, and professional organizations have published evidence-based guidelines and/or implementation aids regarding the prevention of CLABSI including the following:
  - Healthcare Infection Control Practices Advisory Committee (HICPAC), Centers for Disease Control and Prevention (CDC)^52,53^
  - Institute for Healthcare Improvement (IHI)^54^
  - Agency for Healthcare Research and Quality, *Making Health Care Safer*^55^
  - American Pediatric Surgical Association, *Outcomes and Clinical Trials Committee*^56^
  - The Joint Commission^57^
  - APIC, *Implementation Guide to Preventing Central Line-Associated Bloodstream Infections*^58^
  - Infusion Nurses Society, *Infusion Nursing Standards of Practice*^59^
2. The recommendations in this document focus on CVCs unless noted otherwise. These recommendations:
  - Are not stratified based on the type of catheter (eg, tunneled, implanted, cuffed, non-cuffed catheter, dialysis catheter).
  - May not be applicable in their entirety for prevention of bloodstream infections with other intravascular devices.

### Infrastructure requirements

Facilities undertaking CLABSI interventions should have the following elements in place:

1. An adequately staffed infection prevention and control program responsible for identifying patients who meet the surveillance definition for CLABSI.
2. Infection prevention staff and, preferably, information technology support to collect and calculate catheter days as a denominator when computing rates of CLABSI and patient days to allow calculation of CVC utilization. Catheter days from information systems should be validated against a manual method, with a margin of error no greater than ±5%.^60–62^
3. Resources to provide appropriate education and training.
4. Adequate laboratory support for timely processing of specimens and reporting of results, as specified by the supervisor of the surveillance program.

## Section 4: Recommended strategies to prevent CLABSI

Recommendations are categorized as either (1) essential practices that should be adopted by all acute-care hospitals or (2) additional approaches that can be considered in locations and/or populations within hospitals when CLABSIs are not controlled by use of essential practices. Essential practices include recommendations in which the potential to affect CLABSI risk clearly outweighs the potential for undesirable effects. Additional approaches include recommendations in which the intervention is likely to reduce CLABSI risk but there is concern about the risks for undesirable outcomes, recommendations for which the quality of evidence is low, recommendations in which cost-to-benefit ratio may be high, or recommendations in which evidence supports the impact of the intervention in select settings (eg, during outbreaks) or for select patient populations. Hospitals can prioritize their efforts by initially focusing on implementation of the prevention strategies listed as essential practices. If CLABSI surveillance or other risk assessments suggest ongoing opportunities for improvement, hospitals should consider adopting some or all of the prevention approaches listed as additional approaches. These can be implemented in specific locations or patient populations or can be implemented hospital-wide, depending on outcome data, risk assessment, and/or local requirements. Each infection prevention recommendation is given a quality of evidence grade (see Table 2).

### Essential practices for preventing CLABSI recommended for all acute-care hospitals

Some of the following measures have been combined into a “prevention bundle” that focuses on catheter insertion.^63,64^ Numerous studies have documented that use of such bundles is effective, sustainable, and cost-effective in both adults and children.^63,65–68^ Bundles are most likely to be successful if implemented in a previously established patient safety culture and their success depends on adherence to individual measures.^69^ However, data suggests that not all components of bundles may be necessary to achieve an effect on CLABSI rates.^70^ After catheter insertion, maintenance bundles have been proposed to ensure optimal catheter care.^71^ More data are needed to determine which components of the maintenance bundle are essential in reducing risk.^72,73^

#### Before insertion

1. **Provide easy access to an evidence-based list of indications for CVC use to minimize unnecessary CVC placement** (Quality of Evidence: LOW)
2. **Require education and competency assessment of healthcare personnel (HCP) involved in insertion, care, and maintenance of CVCs about CLABSI prevention** (Quality of Evidence: MODERATE)^74–78^
  - Include the indications for catheter use, appropriate insertion and maintenance, the risk of CLABSI, and general infection prevention strategies.
  - Ensure that all HCP involved in catheter insertion and maintenance complete an educational program on essential practices to prevent CLABSI before performing these duties.^79,80^ Periodic retraining with a competency assessment may be of benefit.^81^
  - Periodically assess HCP knowledge of and adherence to preventive measures.
  - Require all HCP who insert a CVC to undergo a credentialing process (as established by the individual healthcare institution) to ensure their competency before independently inserting a CVC and aseptic technique for accessing and maintaining the CVC thereafter.
  - Re-educate when an institution changes components of the infusion system that requires a change in practice (eg, when an institution’s change of the needleless connector requires a change in nursing practice).
  - Use simulation training for proper catheter insertion and maintenance if available.^82–85^
3. **Bathe ICU patients >2 months of age with a chlorhexidine preparation on a daily basis** (Quality of Evidence: HIGH)^86–90^
  - In long-term acute-care hospitals (LTACHs), daily chlorhexidine bathing may also be considered as a preventive measure.^91^
  - The role of chlorhexidine bathing in non-ICU patients remains unclear.^92,93^ One cluster-randomized study found a significant reduction in device-associated bacteremia with CHG bathing in this patient population^93^; however, some of these patients also received methicillin-resistant *Staphylococcus aureus* (MRSA) decolonization, making it difficult to draw firm conclusions regarding CHG bathing alone. Several studies have suggested benefit among adult hematology-oncology patients; however, a similar reduction was not observed for pediatric patients with similar conditions.^94,95^ Accordingly, potential benefits and risks, such as increases in resistance and cost, need to be carefully considered.
  - The safety and efficacy of routine use of chlorhexidine bathing in infants <2 months of postnatal age remains unclear.^96^ Although life-threatening skin injuries from CHG have been reported in very young or very preterm infants, they typically occur in infants with a birthweight <1,000 g who are <7 days postnatal age, and they appear rare in older infants.^97–99^
  - Widespread use of chlorhexidine may be associated with decreased chlorhexidine susceptibility, although the clinical relevance of this finding is not well defined.^100^

#### At insertion

1. **In ICU and non-ICU settings, a facility should have a process in place, such as a checklist, to ensure adherence to infection prevention practices at the time of CVC insertion** (Quality of Evidence: MODERATE)^101^
  - Ensure and document adherence to aseptic technique
    - Checklists have been suggested to ensure optimal insertion practices. If used, the documentation should be done by someone other than the inserter.
    - Observation of CVC insertion should be done by a nurse, physician, or other HCP who has received appropriate education (see above) to ensure that aseptic technique is maintained.
    - HCP should be empowered to stop the procedure if breaches in aseptic technique are observed.
2. **Perform hand hygiene prior to catheter insertion or manipulation** (Quality of Evidence: MODERATE)^102–107^
  - Use an alcohol-based waterless product or soap and water.
    - Use of gloves does not obviate hand hygiene.
3. **The subclavian site is preferred to reduce infectious complications when the catheter is placed in the ICU setting** (Quality of Evidence: HIGH)^33,37,108–110^
  - In the non-ICU setting, the risk of infection between the different sites remains unclear. Importantly, in emergent settings, ensuring life-saving vascular access in the fastest possible way may determine the choice of access site.
  - In children and infants, femoral vein catheterization may be considered if upper body sites are contraindicated.^111^ Tunneled femoral vein catheters, with an exit site outside the diaper area in the mid-thigh, may be safer and provide additional risk reduction.^112,113^
  - Controversy exists regarding infectious and noninfectious complications associated with different short-term CVC access sites.^33^ The risk and benefit of different insertion sites must be considered on an individual basis with regard to infectious and noninfectious complications.^33^ Among others, this applies to patients currently receiving or likely to require hemodialysis in whom the subclavian site is avoided due to risk of stenosis.
  - Do not use peripherally inserted central venous catheters (PICCs) as a strategy to reduce the risk of CLABSI. Risk of infection with PICCs in hospitalized patients approaches that of other CVCs.^114^ However, the majority of CLABSIs due to PICCs occur in non-ICU settings.^115^
  - Midline catheters are increasingly being used as an alternative to CVCs for short-term vascular access, with some observational studies suggesting lower bloodstream infection risk associated with midline catheters versus PICCs^116^ and versus CVCs,^117^ respectively. Randomized controlled trials comparing the risk of bloodstream infections and other complications associated with these devices are needed.
4. **Use an all-inclusive catheter cart or kit** (Quality of Evidence: MODERATE)^118^
  - A catheter cart or kit that contains all necessary components for aseptic catheter insertion should be available and easily accessible in all units where CVCs are inserted.
5. **Use ultrasound guidance for catheter insertion** (Quality of Evidence: HIGH)^119,120^
  - Ultrasound-guided internal jugular and femoral vein catheterization reduces the risk of noninfectious complications associated with CVC placement^121^ but the use of ultrasound may lead to a breach in aseptic technique.^122^
  - It is unclear whether ultrasound-guided subclavian vein insertion reduces risk of infectious complications.
6. **Use maximum sterile barrier precautions during CVC insertion** (Quality of Evidence: MODERATE)^123–128^
  - Use maximum sterile barrier precautions:
    - A mask, cap, sterile gown, and sterile gloves are to be worn by all HCP involved in the catheter insertion procedure.
    - The patient is to be covered with a large (“full-body”) sterile drape during catheter insertion.
  - These measures should also be followed when exchanging a catheter over a guidewire.
  - A prospective, randomized study in surgical patients showed no additional benefit for maximum sterile barrier precautions^126^; nevertheless, most available evidence suggests risk reduction with this intervention.
7. **Use an alcoholic chlorhexidine antiseptic for skin preparation** (Quality of Evidence: HIGH)^42,129–134^
  - Before catheter insertion, apply an alcoholic chlorhexidine solution containing at least 2% chlorhexidine gluconate to the insertion site.
    - The antiseptic solution must be allowed to dry before making the skin puncture.
    - Alcoholic chlorhexidine for skin antisepsis to prevent CLABSI in NICU patients should be used when the benefits are judged to outweigh potential risk.

#### After insertion

1. **Ensure appropriate nurse-to-patient ratio and limit use of float nurses in ICUs** (Quality of Evidence: HIGH)^34,35^
  - Observational studies suggest that an adequate nurse-to-patient ratio must be maintained in ICUs where nurses are managing patients with CVCs and that the number of float nurses working in the ICU environment should be minimized.
2. **Use chlorhexidine-containing dressings for CVCs in patients over 2 months of age** (Quality of Evidence: HIGH)^45,135–142^
  - It is unclear whether there is additional benefit with use of a chlorhexidine-containing dressing if daily chlorhexidine bathing is already established and vice-versa.
  - For long-term catheters (eg, hemodialysis catheters) in well-healed access sites, it is unclear whether use of a chlorhexidine dressing reduces risk of infectious complications.^140,143,144^
  - For children under 2 months of age, use of chlorhexidine dressings remains unclear, particularly in very preterm or low birthweight infants.^98^
3. **For nontunneled CVCs in adults and children, change transparent dressings and perform site care with a chlorhexidine-based antiseptic at least every 7 days or immediately if the dressing is soiled, loose, or damp. Change gauze dressings every 2 days or earlier if the dressing is soiled, loose, or damp.** (Quality of Evidence: MODERATE)^145–148^
  - Less frequent, clinically indicated dressing changes may be used for NICU patients or others at high risk of serious complications from catheter dislodgement.^149^
  - If there is excessive bleeding or drainage from the catheter exit site, use gauze dressings instead of transparent dressings until drainage resolves.
4. **Disinfect catheter hubs, needleless connectors, and injection ports before accessing the catheter** (Quality of Evidence: MODERATE)^150–154^
  - Before accessing catheter hubs, needleless connectors, or injection ports, vigorously apply mechanical friction with an alcoholic chlorhexidine preparation, or 70% alcohol. Alcoholic chlorhexidine may have additional residual activity compared to alcohol for this purpose and is therefore preferred.^155^
  - Apply mechanical friction for a minimum of 5 seconds to reduce contamination.^156,157^ It is unclear whether this duration of disinfection can be generalized to needleless connectors not tested in these studies.
  - Monitor compliance with hub-connector-port disinfection because approximately half of such catheter components are colonized under conditions of standard practice.^152,156,158^
5. **Remove nonessential catheters** (Quality of Evidence: MODERATE)
  - Assess the need for continued intravascular access on a daily basis during multidisciplinary rounds. Remove catheters not required for patient care. Decreasing CVC utilization reduces CRBSI risk.^159^ However, reducing CVC utilization may result in increased use of other intravascular catheters with corresponding infection risk.
  - Audits to determine whether CVCs are routinely removed after their intended use may be helpful.^160,161^ Both simple and multifaceted interventions are effective at reducing unnecessary CVC use.^162,163^
6. **Routine replacement of administration sets not used for blood, blood products, or lipid formulations can be performed at intervals up to 7 days** (Quality of Evidence: HIGH)^164^
  - The optimal replacement of intermittently used administration sets is unresolved.
7. **Perform surveillance for CLABSI in ICU and non-ICU settings** (Quality of Evidence: HIGH)^13,165,166^
  - Measure unit-specific incidence of CLABSI (eg, CLABSI per 1,000 catheter days) and report the data on a regular basis to the units, physician and nursing leadership, and hospital administrators overseeing the units.
  - Compare CLABSI incidence to historical data for individual units and to national rates (ie, NHSN).^167^
  - Audit surveillance as necessary to minimize variation in interobserver reliability.^48,168^

### Additional approaches for preventing CLABSI

Several additional approaches are currently available for use. Perform a CLABSI risk assessment before considering implementation of any of these approaches, taking potential adverse events and costs into consideration. Although it is reasonable to evaluate the utility of technology-based interventions when CLABSI rates are above the institutional- or unit-based threshold, this is also an opportunity to review practices and consider behavioral changes that may be instituted to reduce CLABSI risk. These additional approaches are recommended for use in locations and/or populations within the hospital with unacceptably high CLABSI rates despite implementation of the essential CLABSI prevention strategies listed above. These measures may not be indicated if institutional goals have been consistently achieved.

1. **Use antiseptic- or antimicrobial-impregnated CVCs** (Quality of Evidence: HIGH in adult patients^38,39,169–171^ and MODERATE in pediatric patients^172,173^)
  - The risk of CLABSI is reduced with some currently marketed antiseptic-impregnated (eg, chlorhexidine-silver sulfadiazine) catheters and antimicrobial-impregnated (eg, minocycline-rifampin) catheters. Use such catheters under the following conditions:
    - Hospital units or patient populations have a CLABSI rate above institutional goals despite compliance with essential CLABSI prevention practices. Some evidence suggests that use of antimicrobial CVCs, along with other preventive technologies, may have no additional benefit in patient care units that have already established a low incidence of catheter infections.^174,175^
    - Patients have limited venous access and a history of recurrent CLABSI.
    - Patients are at heightened risk of severe sequelae from a CLABSI (eg, patients with recently implanted intravascular devices such as a prosthetic heart valve or aortic graft).
  - Monitor patients for adverse effects such as anaphylaxis.^176^
  - Many studies investigating antimicrobial-impregnated catheters were performed before infection preventive bundles were routine. Whether such catheters have an impact on CLABSI in such settings remains unknown.
2. **Use antimicrobial lock therapy for long-term CVCs** (Quality of Evidence: HIGH^)177–184^
  - Antibiotic and antiseptic locks are created by filling the lumen of the catheter with a supratherapeutic concentration of an antibiotic solution and leaving the solution in place until the catheter hub is re-accessed. Such an approach can reduce the risk of CLABSI. The optimal antimicrobial agent or combination of agents, their concentration, and duration of lock therapy are matters of ongoing research. Due to concerns regarding the potential for the emergence of resistance in exposed organisms, use antimicrobial locks as a preventative strategy for the following:
    - Patients with long-term hemodialysis catheters who have a history of recurrent CLABSI.^185^
    - Prophylaxis for patients with limited venous access and a history of recurrent CLABSI.
    - Patients who are at heightened risk of severe sequelae from a CLABSI (eg, patients with recently implanted intravascular devices such as a prosthetic heart valve or aortic graft).
  - To minimize systemic toxicity, aspirate rather than flush the antimicrobial lock solution after the dwell time has elapsed.^186–189^ The potential of adverse effects associated with ethanol locks should be carefully considered before use.^190,191^
3. **Use recombinant tissue plasminogen activating factor (rt-PA) once weekly after hemodialysis in patients undergoing hemodialysis through a CVC** (Quality of Evidence: HIGH)^192^
4. **Utilize infusion or vascular access teams for reducing CLABSI rates** (Quality of Evidence: LOW)^193,194^
  - Studies have shown that an infusion/vascular access team responsible for insertion and maintenance of *peripheral* intravenous catheters reduces the risk of bloodstream infections^195^; however, few studies have been performed regarding the impact of intravenous therapy teams on CLABSI rates.^196^
5. **Use antimicrobial ointments for hemodialysis catheter insertion sites** (Quality of Evidence: HIGH)^197–201^
  - Apply polysporin “triple” (where available) or povidone-iodine ointment to hemodialysis catheter insertion if compatible with the catheter material.
  - Ingredients in ointments may interact with the chemical composition of some catheters. Thus, ensure the selected ointment will not interact with the catheter material before any such product is applied to the catheter insertion/exit site. For example, ointments containing glycol should not be applied to insertion/exit sites of polyurethane catheters.
  - Mupirocin ointment should not be applied to the catheter insertion site due to the risks of facilitating mupirocin resistance and the potential damage to polyurethane catheters.
6. **Use an antiseptic-containing hub/connector cap/port protector to cover connectors** (Quality of Evidence: MODERATE)^202–208^
  - The utility of routinely disinfecting hub connectors and ports when using antiseptic-containing hub/connector cap/port protectors is unknown.

#### Approaches that should not be considered a routine part of CLABSI prevention

1. **Do not use antimicrobial prophylaxis for short-term or tunneled catheter insertion or while catheters are in situ** (Quality of Evidence: HIGH)^209–213^
  - Systemic antimicrobial prophylaxis is not recommended.
2. **Do not routinely replace CVCs or arterial catheters** (Quality of Evidence: HIGH)^214^
  - Routine catheter replacement is not recommended.

#### Unresolved issues

1. **Routine use of needleless connectors as a CLABSI prevention strategy before an assessment of risks, benefits, and education regarding proper use**^215–219^
  - Multiple devices are currently available but the optimal design for preventing infections is unresolved. The original purpose of needleless connectors was to prevent needlestick injuries during intermittent use. No data are available regarding their use with continuous infusions. Needle-free connectors with 3-way stopcocks may increase the risk of catheter infections.^220^
    - Use of silver-coated catheter connectors may be associated with reduced intraluminal contamination in ex vivo catheters and CLABSI.^221,222^ Clinical evidence is limited regarding the risk reduction with their routine use or use of other antimicrobial catheter connectors.
2. **Surveillance of other types of catheters (eg, peripheral arterial or venous catheters)**^11,21,22^
  - Peripheral arterial catheters, short-term peripheral venous catheters and midline catheters are not included in most surveillance systems although they are associated with risk of bloodstream infection. Future surveillance systems should consider including bloodstream infections associated with these types of catheters.
  - If considering further infection prevention interventions due to concern for an increase in infections, hospitals may want to consider extending their surveillance programs to include all types of catheters used to gauge the size of the problem.
3. **Standard, nonantimicrobial transparent dressings and CLABSI risk**
  - A meta-analysis reported an association between CLABSI and transparent dressing use; however, the source studies for the meta-analysis reporting this association were of low quality.^223^
4. **The impact of using chlorhexidine-based products on bacterial resistance to chlorhexidine**
  - Widespread use of chlorhexidine-based products (eg, use of chlorhexidine bathing, antisepsis, and dressings) may promote reduced chlorhexidine susceptibility.^224^ However, testing for chlorhexidine susceptibility is not standardized. The clinical impact of reduced chlorhexidine susceptibility is unknown.
5. **Sutureless securement**
  - The impact of sutureless securement devices in reducing CLABSI is unknown.^225,226^
6. **Impact of silver zeolite-impregnated umbilical catheters in preterm infants (applicable in countries where it is approved for use in children)**^227^
  - One randomized study suggests that antimicrobial-impregnated umbilical catheters appear to be safe and effective in NICU patients.^228^
7. **Necessity of mechanical disinfection of a catheter hub, needleless connector, and injection port before accessing the catheter when antiseptic-containing caps are being used.**
  - It is unknown whether the application and removal of an antiseptic-containing cap provides the same benefit to reducing risk of CLABSI as manual disinfection. Future research is needed to determine if using such a cap will obviate the need for manual disinfection before accessing a catheter.

## Section 5: Performance measures

### Internal reporting

These performance measures are intended to support internal hospital quality improvement efforts^229,230^ and do not necessarily address external reporting needs.

The process and outcome measures suggested here are derived from published guidelines, other relevant literature, and the opinion of the authors. Report process and outcome measures to senior hospital leadership, nursing leadership, and clinicians who care for patients at risk for CLABSI.

### Process measures (Table 3)

1. **Compliance with CVC insertion guidelines as documented on an insertion checklist**
  - Assess compliance with the checklist in all hospital settings where CVCs are inserted (eg, ICUs, ED, OR, radiology, general patient care units) and assign HCP familiar with CVCs to this task.
  - Documenting compliance using the insertion checklist upholds accountability and compliance with the proper procedure steps and identifies gaps to be mitigated. The Institute for Healthcare Improvement (IHI) provides an example of a central catheter checklist.^231^
  - Documentation of CVC insertion procedures in compliance with appropriate hand hygiene, use of maximal sterile barrier precautions, and use of chlorhexidine-based cutaneous antisepsis of the insertion site:
    - **Numerator:** Number of CVC insertions that have documented the use of all 3 interventions (hand hygiene, maximal barrier precautions, and chlorhexidine-based cutaneous antiseptic use) performed at the time of CVC insertion.
    - **Denominator:** Number of all CVC insertions.
    - Multiply by 100 so that the measure is expressed as a percentage.
2. **Compliance with documentation of daily assessment regarding the need for continuing CVC access.**
  - Measure the percentage of patients with a CVC where there is documentation of daily assessment:
    - **Numerator:** Number of patients with a CVC who have documentation of daily assessment.
    - **Denominator:** Number of patients with a CVC.
    - Multiply by 100 so that the measure is expressed as a percentage.
3. **Simulation of catheter maintenance as an alternative to address HCP competency**^232,233^
  - **Numerator:** Number of HCP properly simulating the aseptic infusion of medications.
  - **Denominator:** Number of HCP simulating the aseptic infusion of medications.
  - Multiply by 100 so that the measure is expressed as a percentage.
4. Device utilization can be evaluated over time to assess any changes. Utilization may be compared at the hospital and unit level. It provides a surrogate for patient exposure risk.^234^ The standardized utilization ratio (SUR) is an NHSN measure that accounts for facility- and location-level factors that may affect device use.
  - SUR: Observed device days divided by predicted device days.

**Table 3.** CLABSI Prevention Process Measures

Assessing Compliance According to Practice	
Use of proper CVC insertion interventions: 1. Hand hygiene 2. Use of maximal sterile barrier precautions 3. Use of chlorhexidine-based cutaneous antisepsis	(Number of CVC insertions that have documented the use of all 3 interventions performed at the time of CVC insertion divided by number of all CVC insertions) ×100 = % properly performed procedures
Documentation of daily assessment regarding patient’s need for continuing CVC access	(Number of CVC insertions with documentation of daily assessment divided by number of patients with CVC) ×100 = % of patients who received daily assessment for continuing need for CVC access
**Assessing Compliance by Simulation**	
Simulation of catheter maintenance to assess HCP competency	(Number of HCP properly simulating aseptic infusion of medications divided by number of HCP simulating the aseptic infusion of medications) ×100 = % of HCP competent in catheter maintenance
**Assessing Device Utilization as a Surrogate for Patient Exposure Risk**	
Standard utilization ratio (SUR)	Number of observed device days divided by number of predicted device days

### Outcome measures (See Table 4)

1. **CLABSI rate:** Use NHSN definitions.
  - **Numerator:** Number of CLABSIs in each unit assessed (using NHSN definitions).
  - **Denominator:** Total number of catheter days in each unit assessed (using NHSN definitions).
  - Multiply by 1,000 so that the measure is expressed as number of CLABSIs per 1,000 catheter days.
2. **Risk adjustment:** Stratify CLABSI rates by type of patient-care unit.^235–237^
  - Report comparisons based on historic data and NHSN data, if available.^167^
  - Use the NHSN device standardized infection ratio (dSIR) to evaluate hospital and unit CLABSI rates.
    - dSIR: Observed CLABSI events divided by predicted CLABSI events based on actual device days.
  - Consider measures that address device risk at the patient population level. A population SIR (pSIR)^238^ accounts for both device SIR and SUR, reflecting both the care of the device, and interventions to reduce utilization.
    - pSIR: Observed CLABSI events divided by predicted CLABSI events based on predicted device days.

**Table 4.** CLABSI Prevention Outcome Measures

Assessing CLABSI Rate	
Using NHSN definitions	(Number of CLABSIs in each unit assessed with NHSN definitions divided by total number of catheter days in each unit assessed using NHSN definitions) ×1,000 = Number of CLABSIs per 1,000 catheter days
**Risk Adjustment**	
*Report comparisons based on historic data and NHSN data, if available.*	
By type of patient-care unit	Device standardized infection ratio (dSIR) = Observed CLABSI events divided by predicted CLABSI events based on actual device days
By the patient population level to reflect the care of the device, and interventions to reduce utilization	Population standardized infection ratio (pSIR) = Observed CLABSI events divided by predicted CLABSI events based on predicted device days

### External reporting

Many challenges exist in providing useful information to consumers and other stakeholders and in preventing unintended consequences of public reporting of HAIs.^239,240^ Recommendations for public reporting of HAIs have been provided by the Healthcare Infection Control Practices Advisory Committee (HICPAC),^241^ the Healthcare-Associated Infection Working Group of the Joint Public Policy Committee,^242^ and the National Quality Forum.^243^

### State and federal requirements

1. Hospitals in states that have mandatory reporting requirements for CLABSI must collect and report the data required by the state.
2. For information on state and federal requirements, contact your state or local health department.

#### External quality initiatives

1. Hospitals that participate in external quality initiatives or state programs must collect and report the data required by the initiative or the program.
2. Problems with interrater reliability may affect comparisons between different institutions.

## Section 6: Implementation of CLABSI prevention strategies

Prevention of CLABSI depends on integrating best practices to reduce the risk of infection and incorporating a culture to support implementation. Hospitals should address technical and socioadaptive components^244^ to CLABSI prevention, including formal training of HCP on indications, placement, and maintenance of devices, in addition to regular assessment of competencies.^245^

One example of a widely used model in the United States, known as the Four Es (ie, engage, educate, execute, and evaluate^246^), involves summarizing evidence, identifying local barriers to implementation, measuring performance, and ensuring that patients receive the infection prevention intervention^247^ by addressing knowledge, critical thinking, behavior and psychomotor skills, as well as attitudes and beliefs of all members of the healthcare team involved with the insertion and care of CVCs.^248,249^ Facilities may consider utilizing tools to promote high-reliability processes (eg, Lean Six Sigma) and to enhance teamwork (eg, Team STEPPS).

### Engage

Historically, efforts have been centered around having a champion to support CLABSI reduction initiatives. Champions are often very effective in initial phases of adoption, but their efforts may not be enough for integration of processes and sustainability.^250^ It is important to engage both frontline and senior leadership champions in the process and outcome improvement plan,^251^ but institutionalizing the work and garnering the support of stakeholder groups facilitates successful, long-lasting results.^252^

### Educate

HCP, patients, and caregivers involved in care of a CVC should be trained in and competent, relative to their role, with the following:

1. Appropriate indications prior to insertion.
2. Use of full barrier precautions at the time of insertion.
3. Daily evaluation of necessity of the device.

### Execute

A standardized competency assessment checklist should be used to assess and document competency of each individual performing CVC insertion and procedures related to care and maintenance (eg, dressing changes).^253–255^ In addition, education of the patient and/or family, as appropriate, is required for all CVC care procedures especially when transfer to an alternative setting (eg, home care, ambulatory setting) is planned.^256,257^

### Evaluate

Evaluation involves both process and outcome measurement.^258^ Multidisciplinary teams should set clear goals and identify the key factors to be measured. It is important for members of the healthcare team to receive feedback on their performance. Feedback should include periodic (eg, monthly, quarterly) communication (eg, e-mail messages, written reports) of process measurement data via posters, reports, or other forms of communication with graphs showing cumulative compliance with process measures.^259–262^ Differences between age groups should also be considered (eg, neonates, pediatrics, and adults).^260,263,264^ Central line data can be used to capture trends over time. The standardized utilization ratio (SUR) provides a method for the hospital’s units to compare themselves to others with similar characteristics. CLABSI events are important to discuss with the different members of the team caring for the patient to have a clear understanding of gaps and ways to mitigate them in the future.
